# Complete genome sequence of *Schlesneria* sp. strain DSM 10557, isolated from the leachate of an industrial compost heap

**DOI:** 10.1128/mra.01070-24

**Published:** 2024-12-31

**Authors:** Ines Friedrich, Anna Klassen, Anja Poehlein, Robert Hertel, Rolf Daniel

**Affiliations:** 1 Department of Genomic and Applied Microbiology, Georg-August University of Göttingen, Göttingen, Germany; SUNY College of Environmental Science and Forestry, Syracuse, New York, USA

**Keywords:** *Schlesneria* DSM 10557, compost, *Schlesneria*, *Planctomycetaceae*

## Abstract

We present the complete genome of *Schlesneria* sp. DSM 10557, isolated from compost heap leakage (leachate). The genome consists of a single chromosome (7,067,087 bp) with a GC content of 56.48%. Genome-based phylogenetic analysis revealed that strain DSM 10557 is most closely related to members of the genus *Schlesneria*.

## ANNOUNCEMENT

The genus *Schlesneria* comprises Gram-negative, ovoid-shaped, facultative aerobic bacteria originally found in Sphagnum-dominated boreal wetlands (1). Here, we report the complete genome sequence of *Schlesneria* sp. DSM 10557 (strain 639, IFAM 3594), isolated by Griepenburg *et al*. (2) from industrial compost heap leachate (2). Strain DSM 10557 was selected for sequencing due to its close similarity to the genus *Schlesneria*, based on the 16S rRNA gene sequence. Until now, the genus was only represented by the type strain *Schlesneria paludicola* MPL7^T^ (1).

The isolate was obtained from the Deutsche Sammlung für Mikroorganismen und Zellkulturen (DSMZ, Braunschweig, Germany) and cultured in alkaliphiles *Pirellula* medium (M1PY; DSMZ medium 1437) at 30°C. Genomic DNA was extracted from a stationary-phase culture using the MasterPure complete DNA and RNA purification kit (Epicentre, Madison, WI, USA) following the manufacturer’s recommendations. Illumina paired-end sequencing libraries were prepared using the Nextera XT DNA library preparation kit (Illumina, San Diego, CA, USA) and sequenced on a MiSeq instrument with the reagent kit v3 (2 × 300 bp, 600 cycles), according to the manufacturer’s instructions (Illumina). For NanoPore sequencing, 1.5 µg high-molecular-weight DNA from a separate batch was used directly to prepare the library employing the Ligation Sequencing kit (SQK-LSK109) and the Native Barcode Expansion Kit (EXP-NBD103; barcode 8), following the manufacturer’s protocol (Oxford Nanopore Technologies, Oxford, UK). Sequencing was performed using the MinION Mk1B device with a SpotON flow cell R9.4 and MinKNOW software v1.15.4 for 72 hours, as recommended by the manufacturer (Oxford Nanopore Technologies). Default parameters were used for all software, unless otherwise specified. Albacore v2.3.1 (Oxford Nanopore Technologies) was used for base calling and demultiplexing. The sequencing produced 4,233,119 300 bp Illumina reads and 32,562 Nanopore reads (mean length 18,419 bp). Illumina reads were quality-filtered using Trimmomatic v0.39 (3), and paired reads were merged using FLASH v1.2.11 (4). Nanopore reads were trimmed for adapters and quality-filtered using fastp v0.23.2 (5) with a length cutoff of 10 kb, resulting in 13,704 Nanopore reads with an *N*_50_ value of 31,373  bp. A *de novo* hybrid assembly was performed using Unicycler v0.5.0 (6) in the normal mode. The assembly resulted in a single circular chromosome (7,067,087 bp) exhibiting a GC content of 56.48%. The coverage was calculated using Qualimap v2.2.2 (7), Bowtie 2 v2.2.5 (8), and SAMtools v1.16.1 (9), yielding 317-fold coverage for Illumina reads and 61-fold coverage for Nanopore reads. The genome was reoriented to the standard reference position (*dnaA*) with Dnaapler v0.4.0 (10). Genome annotation was performed using the Prokaryotic Genome Annotation Pipeline v6.8 (11), and the assembly quality was assessed using CheckM2 v1.0.2 (12), as summarized in Table 1. Taxonomic classification using GTDBK-Tk v2.3.2 (13) showed the closest match with *Schlesneria paludicola* MPL7^T^ (GCF_000255655.1), with 76.87% nucleotide sequency identity. A whole genome-based phylogeny of the DSM 10557 genome was performed using the Type Strain Genome Server (TYGS (14); accessed 23 September 2024), revealing close relationships to the *Planctomycetaceae* genera *Gimesia*, *Planctellipticum*, *Planctopirus,* and *Schlesneria*. Digital DNA–DNA hybridization (dDDH) with *S. paludicola* MPL7^T^ was calculated to be 21.1%. These results suggest that strain DSM 10557 represents a new species within the genus *Schlesneria*.

**TABLE 1:** General features of the *Schlesneria* sp. DSM 10557 genome (accession: CP124747)

Feature	Amount
Total number of genes	5,026
Number of protein-coding sequences	4,966
Number of rRNA operons	2
5S rRNA genes	2
16S rRNA genes	2
23S rRNA genes	2
Number of tRNA genes	51
Number of ncRNA genes	3
Genome completeness	99.76%
Genome contamination	1.38%

## Data Availability

This complete genome sequence is available at DDBJ/ENA/GenBank under accession number CP124747. The raw reads have been deposited in the NCBI Sequence Read Archive (SRA) under accession numbers SRX20250453 (Illumina) and SRX20250454 (Nanopore).

## References

[1] Kulichevskaya IS, Ivanova AO, Belova SE, Baulina OI, Bodelier PLE, Rijpstra WIC, Sinninghe Damsté JS, Zavarzin GA, Dedysh SN. 2007. Schlesneria paludicola gen. nov., sp. nov., the first acidophilic member of the order Planctomycetales, from Sphagnum-dominated boreal wetlands. Int J Syst Evol Microbiol 57:2680–2687. doi:10.1099/ijs.0.65157-0 17978240

[2] Griepenburg U, Ward-Rainey N, Mohamed S, Schlesner H, Marxsen H, Rainey FA, Stackebrandt E, Auling G. 1999. Phylogenetic diversity, polyamine pattern and DNA base composition of members of the order Planctomycetales. Int J Syst Evol Microbiol 49:689–696. doi:10.1099/00207713-49-2-689 10319492

[3] Bolger AM, Lohse M, Usadel B. 2014. Trimmomatic: a flexible trimmer for Illumina sequence data. Bioinformatics 30:2114–2120. doi:10.1093/bioinformatics/btu170 24695404 PMC4103590

[4] Magoč T, Salzberg SL. 2011. FLASH: fast length adjustment of short reads to improve genome assemblies. Bioinformatics 27:2957–2963. doi:10.1093/bioinformatics/btr507 21903629 PMC3198573

[5] Chen S, Zhou Y, Chen Y, Gu J. 2018. Fastp: an ultra-fast all-in-one FASTQ preprocessor. Bioinformatics 34:i884–i890. doi:10.1093/bioinformatics/bty560 30423086 PMC6129281

[6] Wick RR, Judd LM, Gorrie CL, Holt KE. 2017. Unicycler: resolving bacterial genome assemblies from short and long sequencing reads. PLoS Comput Biol 13:e1005595. doi:10.1371/journal.pcbi.1005595 28594827 PMC5481147

[7] Okonechnikov K, Conesa A, García-Alcalde F. 2016. Qualimap 2: advanced multi-sample quality control for high-throughput sequencing data. Bioinformatics 32:292–294. doi:10.1093/bioinformatics/btv566 26428292 PMC4708105

[8] Langmead B, Salzberg SL. 2012. Fast gapped-read alignment with Bowtie 2. Nat Methods 9:357–359. doi:10.1038/nmeth.1923 22388286 PMC3322381

[9] Danecek P, Bonfield JK, Liddle J, Marshall J, Ohan V, Pollard MO, Whitwham A, Keane T, McCarthy SA, Davies RM, Li H. 2021. Twelve years of SAMtools and BCFtools. Gigascience 10:giab008. doi:10.1093/gigascience/giab008 33590861 PMC7931819

[10] Bouras G, Grigson SR, Papudeshi B, Mallawaarachchi V, Roach MJ. 2024. Dnaapler: a tool to reorient circular microbial genomes. JOSS 9:5968. doi:10.21105/joss.05968

[11] Zhao Y, Wu J, Yang J, Sun S, Xiao J, Yu J. 2012. PGAP: pan-genomes analysis pipeline. Bioinformatics 28:416–418. doi:10.1093/bioinformatics/btr655 22130594 PMC3268234

[12] Chklovski A, Parks DH, Woodcroft BJ, Tyson GW. 2023. CheckM2: a rapid, scalable and accurate tool for assessing microbial genome quality using machine learning. Nat Methods 20:1203–1212. doi:10.1038/s41592-023-01940-w 37500759

[13] Chaumeil P-A, Mussig AJ, Hugenholtz P, Parks DH. 2022. GTDB-Tk v2: memory friendly classification with the genome taxonomy database. Bioinformatics 38:5315–5316. doi:10.1093/bioinformatics/btac672 36218463 PMC9710552

[14] Meier-Kolthoff JP, Göker M. 2019. TYGS is an automated high-throughput platform for state-of-the-art genome-based taxonomy. Nat Commun 10:2182. doi:10.1038/s41467-019-10210-3 31097708 PMC6522516

